# Initial Therapy, Regimen Change, and Persistence in a Spanish Cohort of Newly Treated Type 2 Diabetes Patients: A Retrospective, Observational Study Using Real-World Data

**DOI:** 10.3390/ijerph17103742

**Published:** 2020-05-25

**Authors:** Aida Moreno-Juste, Beatriz Poblador-Plou, Mercedes Aza-Pascual-Salcedo, Francisca González-Rubio, Sara Malo, Julián Librero López, Victoria Pico-Soler, Eva Giménez Labrador, Sara Mucherino, Valentina Orlando, Enrica Menditto, Alexandra Prados-Torres, Antonio Gimeno-Miguel

**Affiliations:** 1 Servicio Aragonés de Salud (SALUD), 50009 Zaragoza, Spain; aidamorenoj@gmail.com (A.M.-J.); maza@salud.aragon.es (M.A.-P.-S.); franciscagonzalezrubio@gmail.com (F.G.-R.); vpico@salud.aragon.es (V.P.-S.); 2 EpiChron Research Group, Aragon Health Sciences Institute (IACS), IIS Aragón, Miguel Servet University Hospital, 50009 Zaragoza, Spain; bpoblador.iacs@aragon.es (B.P.-P.); emgimenezl.iacs@aragon.es (E.G.L.); sprados.iacs@aragon.es (A.P.-T.); 3 Red de Investigación en Servicios de Salud en Enfermedades Crónicas (REDISSEC), ISCIII, 28222 Madrid, Spain; julian.librero.lopez@navarra.es; 4 Grupo de Trabajo de Utilización de Fármacos de la SemFYC, 50009 Zaragoza, Spain; 5 Department of Preventive Medicine and Public Health, Faculty of Medicine, University of Zaragoza, 50009 Zaragoza, Spain; smalo@unizar.es; 6 Grupo de Investigación en Servicios Sanitarios de Aragón (GRISSA), IIS Aragón, 50009 Zaragoza, Spain; 7 Centro de Investigación Biomédica del Gobierno de Navarra (Navarrabiomed), Universidad Pública de Navarra (UPNA), Complejo Hospitalario de Navarra (CHN), 31008 Pamplona, Spain; 8 CIRFF, Center of Pharmacoeconomics, University of Naples Federico II, 80131 Napoli NA, Italy; sara.mucherino@unina.it (S.M.); valentina.orlando@unina.it (V.O.); 9 Department of Pharmacy, University of Naples Federico II, 80131 Napoli NA, Italy

**Keywords:** adherence, antidiabetics, comorbidity, diabetes, persistence, real-world data, Spain, treatment patterns

## Abstract

The World Health Organization considers the non-adherence to medication a significant issue with global impact, especially in chronic conditions such as type 2 diabetes. We aim to study antidiabetic treatment initiation, add-on, treatment switching, and medication persistence. We conducted an observational study on 4247 individuals initiating antidiabetic treatment between 2013 and 2014 in the EpiChron Cohort (Spain). We used Cox regression models to estimate the likelihood of non-persistence after a one-year follow-up, expressed as hazard ratios (HRs). Metformin was the most frequently used first-line antidiabetic (80% of cases); combination treatment was the second most common treatment in adults aged 40–79 years, while dipeptidyl peptidase-4 inhibitors were the second most common in individuals in their 80s and over, and in patients with renal disease. Individuals initiated on metformin were less likely to present addition and switching events compared with any other antidiabetic. Almost 70% of individuals initiated on monotherapy were persistent. Subjects aged 40 and over (HR 0.53–0.63), living in rural (HR 0.79) or more deprived areas (HR 0.77–0.82), or receiving polypharmacy (HR 0.84), were less likely to show discontinuation. Our findings could help identify the population at risk of discontinuation, and offer them closer monitoring for proper integrated management to improve prognosis and health outcomes.

## 1. Introduction

The term diabetes encompasses a major group of chronic diseases with some of the highest prevalence rates and associated mortality risks, as reported by the World Health Organization (WHO) [1]. Various studies report that the incidence of type 2 diabetes (T2D) increased in most populations until the early 2000s, tending to be more stable or even decreasing after approximately 2005 [2]. Its prevalence in adults (85–95% T2D) has almost doubled between 1980 and 2014 worldwide [3]. The factors potentially contributing to diabetes incidence and prevalence variances are numerous, and could explain the differences among populations and over time. These factors include, but are not limited to, lifestyle behaviors (such as poor diet, insomnia, and low physical activity), obesity, higher life expectancy, and changes on the diagnostic criteria of diabetes during the last years [2,4]. In 2015, 374 million people in the world were estimated to have T2D [5], and it has been estimated that T2D prevalence will increase in the near future [6]. Although roughly 6.6–7.0% of the adult population in Spain has been diagnosed with T2D [7], many patients are unaware of their condition, and its real prevalence could be much higher, almost doubling current estimates in some studies [8].

Diabetes represents a large economic burden for healthcare systems. A contributing factor to its economic impact is the rise in the expenditure of patented, branded medicines [9]. Over the past years, new oral antidiabetic drugs have been developed and introduced in the market, such as sodium-glucose co-transporter 2 (SGLT2) inhibitors, dipeptidyl peptidase-4 inhibitors (DPP-4i), and glucagon-like peptide-1 (GLP-1) receptor agonists, among others. This situation makes it necessary to conduct pharmaco-epidemiological studies to explore their usage patterns in clinical practice (e.g., first-choice drugs for treatment initiation, drug switching, and add-on therapy) and the factors that could influence treatment adherence.

One of the main goals of public health systems is to improve the clinical management of patients with T2D. In this sense, ensuring proper medication adherence should be one of their priorities, as sub-optimal adherence to treatment is associated with lower effectiveness of therapeutic strategies, poorer quality of life, and higher risk of morbidity and mortality [10]. Treatment adherence in developed countries is reported to be around 50% in populations with chronic diseases [11,12]. Medication adherence, defined as the process by which patients take their medication as prescribed, entails three essential elements: initiation, implementation, and discontinuation [10]. The early discontinuation of the dispensed treatment is referred to as non-persistence [10,13]. Thus, it is crucial to identify which factors can lead to sub-optimal persistence in the treatment of T2D, which could be influenced by gender [14], age [15], medication class [16], concomitant polypharmacy [17], comorbidity burden [18], and patient education and beliefs, among others [19].

The increasing availability of real-world data (RWD), routinely collected during the care process from hospital and primary care electronic health records (EHRs), clinical-administrative databases, and pharmacy billing records, represents an opportunity to conduct large-scale population studies to generate real-world evidence in the field of healthcare research. This type of data has already been leveraged to analyze the treatment patterns of diabetes in real-life conditions in countries such as Italy [20], France [21], England, and Wales [22].

In Spain, the EPICHRONIC II project aimed to study the utilization patterns of new oral antidiabetic drugs and the factors related to medication adherence through the use of RWD [23]. The aims of this sub-study of the EPICHRONIC II project were as follows: (i) to describe the utilization patterns of initiation antidiabetics for the management of a Spanish cohort of newly treated T2D patients; (ii) to analyze the changes in therapeutic regimens during the one-year period after treatment initiation (i.e., treatment switching and add-on therapy); and (iii) to investigate the socio-demographic and clinical factors related to non-persistence after a one-year follow-up. The results could clarify whether pharmacologic treatment of T2D is in line with existing clinical guidelines and to identify the individuals who could benefit from a closer monitoring to improve their medication adherence and, therefore, T2D health outcomes.

## 2. Materials and Methods

### 2.1. Study Design and Population

We conducted a retrospective, observational study based on the EpiChron Cohort. This cohort links the demographic and clinical information from EHRs and pharmacy billing records of all the public health system users in the Spanish region of Aragón (i.e., 1,144,816 individuals in 2015). A description of the cohort profile and the data sources was published elsewhere [24]. The Institutional Review Board of Aragón (CEICA) approved the research protocol of this study (PI17/0361) and waived the requirement to obtain informed consent from patients as all the information used was pseudoanonymised.

We studied all the individuals of the cohort aged 15 years and over who received at least one prescription of an antidiabetic drug between 1 October 2013 and 30 September 2014 (enrolment period; *n* = 65,167). The date of the first antidiabetic prescription was defined as the index date. Only subjects who had at least two years of valid data before and one year after the index date were included in the study. The new users of oral antidiabetics were identified as subjects without any recorded prescription during the two previous years to the index date.

**Figure 1:**
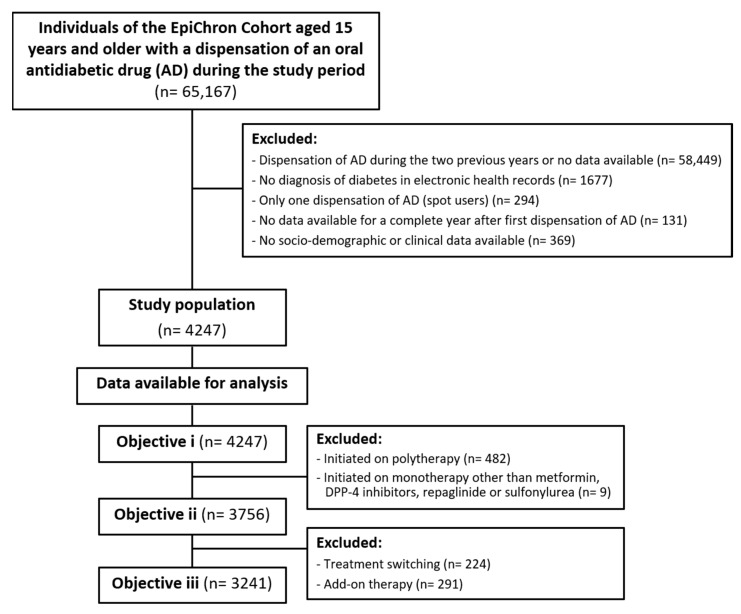
Flow chart of the study population.

We analyzed the antidiabetic agents included in the Anatomical Therapeutic Chemical (ATC) Classification System code *A10B*. New users were classified in different categories based on the first chemical subgroup they received during the enrolment period: metformin (ATC *A10BA*); sulfonylureas (*A10BB*); DPP-4i (*A10BH*); repaglinide (*A10BX*); and other monotherapy treatments including alpha-glucosidase inhibitors (*A10BF*), thiazolidinediones (*A10BG*), or GLP-1 receptor agonists (*A10BJ*). SGLT2 inhibitors were not commercialized during the study period, and thus were not analyzed. Subjects receiving drugs from the ATC code *A10BD* (i.e., combinations of oral blood glucose-lowering drugs) were classified as fixed combination therapy, considering each of their single active agents. Individuals receiving two different drugs with an overlapping period of at least 15 days were classified as free-combination therapy, following the line of previous studies [20,25]. We excluded from the analysis subjects with a single prescription (i.e., spot users), those lacking a T2D diagnosis in their EHRs during the study period, and those in which socio-demographic and clinical information was unavailable (Figure 1). Individuals were followed for 365 days from treatment initiation to analyze the antidiabetics dispensation patterns.

### 2.2. Study Variables and Outcomes

For each subject, we assessed the following socio-demographic variables: age at the index date (i.e., 15–39, 40–59, 60–79, and ≥80 years), gender, administrative health area (urban—those that concentrate in one of its municipalities at least 80% of the population of the area, and rural—the rest), deprivation index of the area according to 26 socio-economic indicators and categorized from least (Q1) to most (Q4) deprived [26], and immigration status (native or immigrant). We also analyzed the number of drugs dispensed simultaneously (referred to as concomitant drugs), the number of comorbidities accompanying T2D (0, 1–4, or ≥5 conditions), and the presence of chronic renal failure. For the assessment of drugs dispensed to the patient, we considered all of the medications except for drugs within the anatomical groups J (systemic anti-infectives) and V (various). Subjects were classified as having no polypharmacy (0–5 drugs), polypharmacy (6–9 drugs), or excessive polypharmacy (≥10 drugs).

Comorbidity diagnoses were extracted from primary care and hospital EHRs and coded according to the International Classification of Primary Care (ICPC) and to the International Classification of Diseases, Ninth Revision, Clinical Modification (ICD-9-CM), respectively. Diagnoses were then grouped in expanded diagnostic clusters (EDCs) using the Johns Hopkins ACG© System (version 11.0, The Johns Hopkins University, Baltimore, MD, US) [27]. For this study, we only considered the 114 EDCs defined as chronic in the study by Salisbury et al. [28], as well as rhinitis (EDC *ALL03*), according to recent WHO indications [29], and acute lower respiratory tract infection (EDC *RES02*), as it can lead to chronic sequelae. Diabetes was not considered in the total count because it was the index disease for all subjects.

We analyzed initiation treatment patterns (objective i) considering all the new users initiated on any antidiabetic drug (*n* = 4247). The evaluation of treatment switching and add-on therapy (objective ii) only included cases initiated on monotherapy treatment with metformin, DPP-4i, repaglinide, or sulfonylurea (*n* = 3756). Treatment switching was defined as the discontinuation of initial antidiabetic drugs followed by the initiation of an alternative agent from a different drug class. Subjects returning to their initial therapy within 15 days of switching were classified according to their initial therapy and not as switchers. Add-on therapy was defined as receiving an antidiabetic drug from a different therapeutic class while continuing on the initial treatment. Each active agent was considered individually for add-on therapy evaluation even when included in fixed combinations of drugs.

For the evaluation of medication persistence (objective iii), we only considered cases initiated on monotherapy with metformin, DPP-4i, repaglinide, or sulfonylurea and without treatment switching or add-on therapy during the follow-up period (*n* = 3241). We defined persistence as continuous treatment dispensation during 365 days from the index date. Medication persistence was assessed at the fourth ATC level (i.e., drug class level). Persistence was estimated by measuring the time gap between consecutive drug dispensations. Subjects were censored if the permitted gap was exceeded without receiving a new prescription or upon reaching the end of the study period (if they had been persistent throughout the follow-up period). The number of days covered by medication supplies was estimated based on the number of defined daily doses (DDDs) per dispensed package, assuming dosages for drugs from each ATC code followed the WHO recommendations; except for repaglinide, for which a dose of three pills per day was assumed. Subjects were considered as non-persistent (i.e., discontinuer) if he/she presented a gap of ≥90 days between two dispensations [30,31,32,33]. Discontinuers were categorized as users who restarted antidiabetic therapy after a period of discontinuation higher than the maximum permissible gap, or users who simply discontinued treatment without receiving any further prescription after the end of the maximum permissible gap.

### 2.3. Statistical Analysis

We conducted a descriptive analysis of our subjects’ characteristics and of the initial AD treatment patterns. Time to treatment switching or add-on was calculated as the median number of days accompanied by the interquartile range (IQR). We used chi-square tests and unpaired t-tests for the comparison of categorical and numerical variables, respectively. Persistence rates were estimated using the Kaplan–Meier method, and we used the log-rank test to assess statistical differences among curves. We used Cox regression models to estimate the likelihood of discontinuation over one year after treatment initiation, based on the studied socio-demographic and clinical variables. In the models, we analyzed the effect of the following variables: age, gender, living area (urban/rural), deprivation index of the area, number of concomitant drugs, number of comorbidities, presence of chronic renal failure, and drugs used for initiation. For the analysis of each variable, the model was controlled for the rest of factors as potential confounders. We calculated crude and adjusted hazard ratios (HRs), accompanied by their respective 95% confidence intervals (CIs). Statistical significance was set at *p* < 0.05. Statistical analyses were conducted using STATA software (Version 12.0, StataCorp LLC, College Station, TX, USA).

## 3. Results

### 3.1. Characteristics of Study Population and Antidiabetic Therapy

The final study population was comprised of 4247 participants with T2D (57.6% men, mean age of 64.6 ± 12.8 years; Table 1). The vast majority of our patients were natives (95% vs. 5.0% immigrants, *p* = 0.025), more than half of them lived in urban areas (58.6%), and one in three (30.3%) lived in the most (Q4) deprived areas. At treatment initiation, most of the individuals were not diagnosed with chronic renal failure (94.4% vs. 5.6%, *p* < 0.001). Only 6.4% of them did not present any other chronic diseases, 59.3% suffered from one to four chronic diseases, and 34.3% had more than five chronic diseases in addition to T2D. More than half of the subjects (51%) had polypharmacy or excessive polypharmacy.

Initial treatment with monotherapy was observed in 88.7% of cases, whereas 11.3% of them received combination therapy at initiation (Table 1). Metformin was the most commonly dispensed monotherapy drug, accounting for 80.5% of prescriptions, followed by DPP-4i (5.2%); repaglinide (1.5%); sulfonylureas (1.2%); and other monotherapy treatments (0.2%) such as alpha glucosidase inhibitors, thiazolidinediones, and GLP-1 analogues. The most frequently dispensed fixed combinations (data not shown) combined metformin with sitagliptin (197 cases), with vildagliptin (126 cases) or with linagliptin (54 cases).

Metformin in monotherapy was the most frequently dispensed drug in all age groups. In patients aged 15–39, metformin, DPP-4i, and combination therapy were the only treatments dispensed. Fixed combination therapies were most used in the group aged 40–59, while the oldest group aged ≥80 received more DPP-4i in monotherapy than any other age group. Combination therapy was more frequently dispensed in men (12.5%), immigrants (17.9%), and subjects from rural (13.1%) and more deprived (Q3, 12.5% and Q4, 12.1%) areas.

New users showed a mean number of 6.2 ± 4.4 concomitant drugs, although the medication burden was much higher in patients who initiated their treatment with DPP-4i (8.2 ± 4.7 drugs) and repaglinide (8.1 ± 5.4 drugs). Another remarkable difference in the pattern of dispensation depending on the number of concomitant drugs was that 14.3%, 8.3%, and 8.7% of patients treated with 0–5, 6–9, and 10 or more concomitant drugs, respectively, received combination therapy.

Similarly, the burden of comorbidity was higher in patients who initiated their treatment with repaglinide (5.1 ± 4.0 diseases) and DPP-4i (5.1 ± 3.1 diseases). An initial treatment based on combination therapy was more frequent in individuals without additional comorbidities to T2D, while DPP-4i was more frequently dispensed in patients with more than five comorbidities (8.0% of the patients). The presence of chronic renal failure at treatment initiation influenced the choice of the antidiabetic treatment; only 51.7% of patients with chronic renal failure received metformin prescriptions, compared with the 82.2% of patients without this disease (*p* < 0.001). The dispensation of DPP-4i, repaglinide, and sulfonylurea was higher in individuals with chronic renal failure.

### 3.2. Changes in Therapy Regimen

During the one-year follow-up, 6.0% of new users of antidiabetics switched to another treatment (Table 2). The initial treatment with more switching was repaglinide (7.7%), closely followed by DDP-4i (7.2%), sulfonylurea (6.0%), and metformin (5.8%). Most treatment switches in patients initiated on metformin were made towards the use of DDP-4i, followed by sulfonylureas and repaglinide. Patients treated with DPP-4i switched mostly to metformin (43.8%), followed by sulfonylurea (25.0%) and repaglinide (25.0%).

Overall, 7.8% of the study population received add-on therapy during follow-up. Among subjects initially treated with metformin, the add-ons mainly included DPP-4i (79.9%), followed by insulin (6.6%) and sulfonylureas (6.3%). Patients initially treated with DPP-4i had metformin and repaglinide added in 75.9% and 17.2% of cases, respectively. Among individuals on sulfonylurea, no additions to the initial treatment were observed, and only six patients on repaglinide treatment received add-on therapy.

### 3.3. Medication Persistence

We included 3241 subjects in the Kaplan–Meier persistence analysis (Table 3); 69.0% of them were still taking their initial treatment after one year from treatment initiation. Persistence rates varied within the different monotherapy treatment groups, being higher for DPP-4i (76.7%), followed by metformin (68.8%), sulfonylureas (63.8%), and repaglinide (61.1%), although these differences were not significant. The median time to discontinuation was 108 days, varying from 79 days in subjects initiated with DPP-4i to 150 days in those with sulfonylureas. Time to treatment switching was (median, IQR) 55 (21–145) days, and time to add-on therapy was 85 (40–200) days, with significant differences among monotherapy treatment groups.

The Cox regression analysis (Table 4) revealed that older patients associated a lower risk of treatment discontinuation, compared with the age group of 15–39 years. Individuals living in rural areas had 21% less risk of being discontinuers than patients living in an urban area, and those living in less deprived (Q1) areas were more likely to be discontinuers than those from Q2–Q4 areas. Subjects with a dispensation of 6–9 drugs presented 16% lower risk of discontinuation compared with those with up to five drugs. The rest of the potential predictors (i.e., gender, number of comorbidities, presence of chronic renal failure, and drug used at initiation) were not significantly associated with treatment persistence, although individuals initiated on DPP-4i were 27% less likely (*p* = 0.052) to be non-persistent compared with those initiated on metformin.

## 4. Discussion

This study showed that electronic health records can be a valuable information source for pharmaco-epidemiological studies. Studies like ours, which a focus on initiation, implementation, and discontinuation in patients with T2D, are of extreme importance, with the potential to provide tools for adherence-enhancing interventions in daily clinical practice [34]. Our results offer a comprehensive overview of the utilization patterns of antidiabetics and the factors affecting medication adherence in a Spanish cohort of new users of antidiabetic drugs from a real-world setting.

Metformin was by far the most frequently used drug in monotherapy at treatment initiation (80.5% of cases), in line with the recommendations of the European Association for the Study of Diabetes [35] and of the American Diabetes Association [36]. The use of metformin as a first-line antidiabetic drug has also been reported in other European countries such as the United Kingdom (80% of cases) [22], Italy (70%) [20], and France (62%) [37]. In contrast, in other countries like Japan, DPP-4i has been reported as the most prevalent outpatient antidiabetic monotherapy, followed by metformin [38]. In our study, metformin was the first line medication choice for patients with chronic renal failure, although with a lower prevalence than in patients without this condition. This was expected, as metformin can be prescribed initially in patients with glomerular filtration rates over 45 mL/min/1.73 m^2^, and the dose of metformin is usually adapted to the patient’s kidney function, as indicated by various clinical guidelines [39,40,41,42]. The use of metformin was followed far behind by DPP-4i, repaglinide, and sulfonylureas. The choice of these alternatives as initial antidiabetic treatments could be related to cases where the use of metformin was contraindicated [36]. For instance, we observed that our oldest individuals and those with renal disease, both at higher risk for lactic acidosis, received DPP-4i more frequently than other groups, which might be partly explained by the perception that DPP-4i is a safer treatment option, with the subsequent avoidance of metformin [38].

Sulfonylureas have been shown to be the second option after metformin in populations from Italy [20], Ireland [43], and Canada [44]. Nonetheless, a recent study comparing antidiabetic treatment patterns among different regions showed that sulfonylurea prescription as a second-line treatment had decreased, as opposed to the substantial increase of DPP-4i prescription from 2007 to 2011 in France, the United Kingdom, and Spain [45]. The limited dispensation of other antidiabetic classes in our study, like GLP-1 receptor agonists and SGLT2 inhibitors, could be partially explained by the fact that we only included newly diagnosed and treated T2D patients, and that not all the classes of GLP-1 receptor agonists were commercialized during the study period. SGLT2 inhibitors were not included in our study because they were not introduced in the Spanish market until after mid-2015.

Combination therapy, which is not recommended at initiation in clinical guidelines except for in patients with high levels of glycated hemoglobin (HbA1c), was dispensed as first-line treatment in 11.3% of our subjects [36]. Individuals aged 40–59 and immigrants were the population groups that most commonly received combination treatment. This could be because of a greater proportion of individuals with HbA1c values ≥9% in these groups, which, according to guidelines, would justify the initiation with combination therapy to more expeditiously achieve the targeted HbA1c values [36].

During the one-year follow-up, 7.8% of the cohort received a second antidiabetic drug after treatment initiation, whereas 6% of subjects switched to another antidiabetic therapy. These events probably responded to the presence of contraindications, side effects, or lack of effectiveness of the first-choice treatment [35,36]. Individuals initiated on metformin were less likely to present addition and switching events compared with those initiated on any other alternative antidiabetic drug. The lower incidence of treatment adjustments in patients on metformin suggests that using this drug at initiation could prove beneficial in reducing the risk of suboptimal glycemic control and/or of adverse drug events compared with other antidiabetic classes [46]. Some individuals initiated on metformin received add-on insulin while maintaining metformin, in agreement with the recommendations set by guidelines when treatment intensification is needed [35,36].

Despite current recommendations on the use of metformin as a first-line antidiabetic, unless clinically contraindicated [35,36], a noteworthy proportion of patients receiving alternative oral agents at initiation eventually switched to metformin or had it added to their basal treatments. In line with previous studies [38], DPP-4i represented the most frequent add-on and switch treatments. DPP-4i could be preferable in cases where metformin is contraindicated or not tolerated, or in cases with a higher risk of hypoglycemia associated with the use of sulfonylureas [35].

The high persistence rate found in our study (almost 70% of cases) is consistent with the 41–81% reported in a recent meta-analysis [47] and similar to the 79% showed by Italian [20] and Canadian populations [48], although these last two studies included treatment switching and add-on therapy cases in the persistence analysis. The four monotherapy treatments analyzed in our study did not show significant differences regarding persistence rates. Nonetheless, individuals initiated on DPP-4i presented 27% lower risk of discontinuation after 12 months of treatment, in line with previous studies reporting higher persistence in DPP-4i users compared with those using the rest of the antidiabetic drug classes [49,50].

Treatment persistence is a result of several determining factors including effectiveness, tolerability, safety, superior utility, and treatment costs [50]. Regardless of the drug used, prescribing physicians might also play an important role in medication persistence in relation to their empathy with the patient and the type and quality of the information given to him/her. However, this factor could not be included in our analyses. Cox regression analysis showed that subjects aged 40 and over (compared with younger individuals), those living in rural and more deprived areas, and those receiving polypharmacy were less likely to discontinue their initial antidiabetic therapy after one year post-initiation.

There is no consensus in the existing literature on the influence of age on treatment discontinuation [51]. Adherence has been reported to increase with age in some studies [49,52], whereas the opposite has also been observed in other published works [4,20,51,53]. The effect of concomitant polypharmacy on medication adherence is also controversial. We observed that polymedicated patients receiving 6–9 simultaneous drugs were less likely to be discontinuers than non-polymedicated ones. A plausible explanation for this could be that patients with a greater number of comorbid conditions might be more knowledgeable in diabetes and its complications, which would encourage them to continue their diabetes treatment regimens [54]. However, a negative influence on medication adherence of polypharmacy has also been observed [20,49,51]. The effect of comorbidity on medication adherence in diabetic patients is also inconsistent across the existing literature [52].

The relationship found in our study between living area and the risk of treatment discontinuation is consistent with previous studies. Higher non-adherence risks in patients living in urban compared with rural areas have also been observed in Italian [20] and Canadian [48,55] populations. The risk of treatment discontinuation could be related to higher levels of anxiety, increased consumption of processed foods, lower physical activity, and less sleep time, typical of urban areas [56]. Data suggest that regular control, perception of long-term treatment benefit, reduction of treatment complexity, use of preparations with minimal adverse effects, and appropriate re-imbursement could greatly increase persistence in oral antidiabetic therapies [57].

### Strengths and Limitations

The main strength of this study is its large sample size, which included almost all T2D patients in the reference population initiating oral antidiabetic treatment. Furthermore, to the best of our knowledge, this is the first study of its kind conducted in Spain. We extracted all our variables from patient EHRs and pharmacy billing records, making the analyses more reliable and accurate than when using self-reported information. In this regard, cohort data underwent continuous quality control checks to ensure their rigor for research purposes. In the analysis of factors associated with non-persistence, we performed a comprehensive adjustment for covariates.

One of the most important limitations of the study lies in the absence of a standard method to assess medication persistence (e.g., time gap, definition of new user), which compels us to be cautious in the interpretation and comparison of results to those obtained in other studies. Although the measurement method for the estimation of medication persistence had been previously validated [20,54], persistence could have been overestimated in patients who picked up their medication from the pharmacy, but did not take it at home. Another limitation lies in the unavailability of variables that could have been of specific interest for the study, such as lifestyle habits (e.g., smoking and drinking behavior, diet quality, level of physical exercise), glycated hemoglobin, C-peptide values, and glomerular filtration rates, or the totality of drugs prescribed (and not only those finally dispensed to the patient). It would also have been interesting to analyze dose changes over the first year, but this was impossible as information on drug dosage was unavailable. Thus, a comprehensive assessment of predictors for treatment discontinuation, switching, or add-on was not viable. Moreover, SGLT2 inhibitors were not included as they were unavailable in the market during the study period. Moreover, our results could have been different if we had used a longer follow-up period, as we know that the drug utilization pattern can vary over time in a population owing to factors related to prescribers, patients, the pharmaceutical industry, or the social and political contexts. Nonetheless, this study sheds light on the patterns of antidiabetic drugs in new users, as well as on the factors associated with a greater or lesser probability of treatment discontinuation, so that our results could contribute to increase our knowledge in the field.

## 5. Conclusions

Diabetes is a disease with a high clinical and social impact; therefore, its proper management and control is a priority for health systems. This study provides real-world evidence that the utilization pattern of oral antidiabetic drugs in T2D patients in Spain is consistent with the recommendations of international clinical guidelines. Our findings might help identify individuals in which medication persistence shows room for improvement (i.e., younger patients and those living in urban/less deprived areas). These patients could benefit from a closer monitoring of their antidiabetic treatment from primary care to improve treatment effectiveness and glycemic control, and reduce the likelihood of chronic complications. The implementation of person-centered approaches is crucial, especially by giving patients the opportunity to better understand what diabetes is, how it evolves over time, and the importance of following clinical recommendations. Continuous educational activities within a multidisciplinary, integrated management program of T2D patients could potentially lead to better health outcomes.

## Figures and Tables

**Table 1:** Demographic and clinical characteristics of new users of oral antidiabetic drugs at cohort entry (objective i).

Characteristics	Monotherapy					Combination Therapy		Total	*p*-Value ^c^
	Metformin	DPP-4i ^a^	Repaglinide	Sulfonylureas	Other ^b^	Fixed	Free		
**N** (%)	3420 (80.5%)	221 (5.2%)	65 (1.5%)	50 (1.2%)	9 (0.2%)	354 (8.3%)	128 (3.0%)	4247 (100%)	
**Age** (years)									
Mean ± SD ^d^	64.2 ± 12.6	71.6 ± 13.3	72.9 ± 11.8	71.6 ± 14.3	77.6 ± 5.9	61.6 ± 12.5	64.2 ± 11.8	64.6 ± 12.8	<0.001
Age interval (*n*, %)									
15–39	99 (85.3%)	5 (4.3%)	0 (0.0%)	0 (0.0%)	0 (0.0%)	10 (8.6%)	2 (1.7%)	116 (100%)	
40–59	1135 (81.6%)	35 (2.5%)	14 (1.0%)	12 (0.9%)	0 (0.0%)	152 (10.9%)	43 (3.1%)	1391 (100%)	
60–79	1750 (82.3%)	103 (4.8%)	27 (1.3%)	18 (0.9%)	6 (0.3%)	158 (7.4%)	64 (3.0%)	2126 (100%)	
≥80	436 (71.0%)	78 (12.7%)	24 (3.9%)	20 (3.3%)	3 (0.5%)	34 (5.5%)	19 (3.1%)	614 (100%)	
**Gender** (*n*, %)									0.079
Women	1468 (81.5%)	101 (5.6%)	33 (1.8%)	20 (1.1%)	5 (0.3%)	128 (7.1%)	47 (2.6%)	1802 (100%)	
Men	1952 (79.8%)	120 (4.9%)	32 (1.3%)	30 (1.2%)	4 (0.2%)	226 (9.2%)	81 (3.3%)	2445 (100%)	
**Residence area** (*n*, %)									0.008
Urban	2027 (81.5%)	140 (5.6%)	31 (1.3%)	34 (1.4%)	4 (0.2%)	190 (7.6%)	62 (2.5%)	2488 (100%)	
Rural	1393 (79.2%)	81 (4.6%)	34 (1.9%)	16 (0.9%)	5 (0.3%)	164 (9.3%)	66 (3.8%)	1759 (100%)	
**Depriv. index ^e^** (*n*, %)									0.295
Q1	798 (79.5%)	56 (5.6%)	16 (1.6%)	15 (1.5%)	2 (0.2%)	83 (8.3%)	34 (3.4%)	1004 (100%)	
Q2	893 (83.1%)	56 (5.2%)	17 (1.6%)	6 (0.6%)	3 (0.3%)	68 (6.3%)	32 (3.0%)	1075 (100%)	
Q3	692 (78.6%)	48 (5.5%)	18 (2.1%)	11 (1.3%)	1 (0.1%)	81 (9.2%)	29 (3.3%)	880 (100%)	
Q4	1037 (80.5%)	61 (4.7%)	14 (1.1%)	18 (1.4%)	3 (0.2%)	122 (9.5%)	33 (2.6%)	1288 (100%)	
**Immigrant status** (*n*, %)									0.025
Native	3266 (80.7%)	214 (5.3%)	63 (1.6%)	48 (1.2%)	9 (0.2%)	331 (8.2%)	115 (2.8%)	4046 (100%)	
Immigrant	154 (76.6%)	7 (3.5%)	2 (1.0%)	2 (1.0%)	0 (0.0%)	23 (11.4%)	13 (6.5%)	201 (100%)	
**Concomitant drugs**									
Mean ± SD	6.2 ± 4.2	8.2 ± 4.7	8.1 ± 5.4	5.9 ± 4.1	5.9 ± 3.1	5.1 ± 4.6	4.5 ± 4.6	6.2 ± 4.4	<0.001
0–5	1661 (79.8%)	65 (3.1%)	25 (1.2%)	29 (1.4%)	4 (0.2%)	218 (10.5%)	80 (3.8%)	2082 (100%)	
6–9	1051 (83.8%)	75 (6.0%)	13 (1.0%)	8 (0.6%)	3 (0.2%)	72 (5.7%)	33 (2.6%)	1255 (100%)	
≥10	708 (77.8%)	81 (8.9%)	27 (3.0%)	13 (1.4%)	2 (0.2%)	64 (7.0%)	15 (1.7%)	910 (100%)	
**Comorbidities**									
Mean ± SD	3.9 ± 2.6	5.1 ± 3.1	5.1 ± 4.0	4.0 ± 2.5	3.4 ± 1.7	3.3 ± 3.0	3.0 ± 2.4	3.9 ± 2.7	<0.001
0	199 (73.4%)	6 (2.2%)	4 (1.5%)	3 (1.1%)	0 (0.0%)	45 (16.6%)	14 (5.2%)	271 (100%)	
1–4	2046 (81.2%)	98 (3.9%)	33 (1.3%)	29 (1.2%)	7 (0.3%)	218 (8.7%)	88 (3.5%)	2519 (100%)	
≥5	1175 (80.7%)	117 (8.0%)	28 (1.9%)	18 (1.2%)	2 (0.1%)	91 (6.3%)	26 (1.8%)	1457 (100%)	
**Chronic renal failure**									<0.001
No	3297 (82.2%)	166 (4.1%)	44 (1.1%)	39 (1.0%)	8 (0.2%)	335 (8.4%)	120 (3.0%)	4009 (100%)	
Yes	123 (51.7%)	55 (23.1%)	21 (8.8%)	11 (4.6%)	1 (0.4%)	19 (8.0%)	8 (3.4%)	238 (100%)	

^a^ DPP-4i, dipeptidyl peptidase-4 inhibitors; ^b^ other monotherapy: alpha glucosidase inhibitors (ATC *A10BF*), thiazolidinediones (ATC *A10BG*), and glucagon-like peptide-1 (GLP-1) receptor agonists (ATC *A10BJ*); ^c^ *p*-value less than 0.05 was statistically significant; ^d^ standard deviation; ^e^ deprivation index of the area calculated according to 26 socio-economic indicators and categorized from least (Q1) to most (Q4) deprived.

**Table 2:** Treatment switching and add-on therapy patterns among new users of antidiabetic drugs initiated on monotherapy (objective ii).

Initial Therapy	Total	Type of Treatment Switching/Add-On Therapy (*n*, % ^b^)						
	(*n*, % ^a^)	Metformin	DPP-4i ^c^	Repaglinide	Sulfonylureas	Other ^d^	Polytherapy	Insulin
**Total** (*n* = 3756)								
Switchers	224 (6.0)	9 (4.0)	128 (57.1)	25 (11.2)	47 (20.8)	1 (0.4)	9 (4.0)	5 (2.2)
Add-on	291 (7.7)	25 (8.6)	204 (70.1)	17 (5.8)	16 (5.5)	3 (1.0)	6 (2.1)	20 (6.9)
**Metformin** (*n* = 3420)								
Switchers	200 (5.8)	-	127 (63.5)	21 (10.5)	43 (21.5)	1 (0.5)	5 (2.5)	3 (1.5)
Add-on	256 (7.5)	-	204 (79.7)	12 (4.7)	16 (6.3)	2 (0.8)	5 (2.0)	17 (6.6)
**DPP-4i** (*n* = 221)								
Switchers	16 (7.2)	7 (43.8)	-	4 (25.0)	4 (25.0)	0 (0.0)	1 (6.3)	0 (0.0)
Add-on	29 (13.1)	22 (75.9)	-	5 (17.2)	0 (0.0)	1 (3.5)	0 (0.0)	1 (3.5)
**Repaglinide** (*n* = 65)								
Switchers	5 (7.7)	1 (20.0)	1 (20.0)	-	0 (0.0)	0 (0.0)	1 (20.0)	2 (40.0)
Add-on	6 (9.2)	3 (50.0)	0 (0.0)		0 (0.0)	0 (0.0)	1 (16.7)	2 (33.3)
**Sulfonylurea** (*n* = 50)								
Switchers	3 (6.0)	1 (33.3)	0 (0.0)	0 (0.0)	-	0 (0.0)	2 (66.7)	0 (0.0)
Add-on	0 (0.0)	-	-	-	-	-	-	-

^a^ The denominators used for the proportion estimates presented in this column correspond to the figures of the column “Initial therapy”; ^b^ the denominators used for the proportion estimates presented in these rows correspond to the figures of the same row of the column “Total”; ^c^ dipeptidyl peptidase-4 inhibitors; ^d^ other monotherapy: alpha glucosidase inhibitors (ATC *A10BF*), thiazolidinediones (ATC *A10BG*), and glucagon-like peptide-1 (GLP-1) receptor agonists (ATC *A10BJ*).

**Table 3:** Persistence and discontinuation among new users of antidiabetic drugs and time to discontinuation, treatment switching or add-on therapy, by initial monotherapy treatment (objectives ii and iii).

	Initial Monotherapy Treatment					
	Metformin	DPP-4i ^a^	Repaglinide	Sulfonylureas	Total	*p*-Value ^b^
**Frequency** (N, %)						0.068
Persistence	2038 (68.8%)	135 (76.7%)	33 (61.1%)	30 (63.8%)	2236 (69.0%)	
Discontinuation	926 (31.2%)	41 (23.3%)	21 (38.9%)	17 (36.2%)	1005 (31.0%)	
**Days to** (median, IQR ^c^)						
Discontinuation	108 (25–206)	79 (57–195)	133 (30–226)	150 (62–231)	108 (25–206)	0.507
Switching	55 (21–140)	98.5 (33–195)	6 (3–15)	25 (2–73)	55 (21–145)	0.054
Add-on	84 (39–199)	159 (76–217)	31.5 (20–38)	-	85 (40–200)	0.025

^a^ Dipeptidyl peptidase-4 inhibitors; ^b^ *p*-values less than 0.05 were considered statistically significant; ^c^ IQR, interquartile range.

**Table 4:** Predictors of treatment discontinuation to initial antidiabetic therapy at one year post-initiation (objective iii).

Variables	Crude HR ^a^ (95% CI)	*p*-Value	Adjusted HR ^b^ (95% CI)	*p*-Value ^c^
**Age** (years)				
15–39	Reference		Reference	
40–59	0.59 (0.43–0.81)	0.001	0.63 (0.46–0.86)	0.004
60–79	0.49 (0.36–0.67)	<0.001	0.53 (0.38–0.72)	<0.001
≥80	0.50 (0.36–0.71)	<0.001	0.55 (0.39–0.78)	0.001
**Gender**				
Men	Reference		Reference	
Women	1.10 (0.97–1.24)	0.138	1.14 (1.00–1.29)	0.053
**Area of living**				
Urban	Reference		Reference	
Rural	0.77 (0.67–0.87)	<0.001	0.79 (0.69–0.90)	<0.001
**Deprivation index ^d^**				
Q1	Reference		Reference	
Q2	0.77 (0.65–0.91)	0.003	0.79 (0.66–0.94)	0.007
Q3	0.78 (0.65–0.93)	0.007	0.82 (0.68–0.98)	0.031
Q4	0.75 (0.64–0.89)	0.001	0.77 (0.65–0.91)	0.002
**Concomitant drugs**				
0–5	Reference		Reference	
6–9	0.79 (0.68–0.91)	0.001	0.84 (0.72–0.99)	0.034
≥10	0.92 (0.78–1.08)	0.295	1.03 (0.85–1.25)	0.783
**Comorbidities**				
0	Reference		Reference	
1–4	0.91 (0.70–1.19)	0.503	1.02 (0.78–1.34)	0.870
≥5	0.79 (0.60–1.05)	0.103	0.92 (0.68–1.26)	0.611
**Chronic renal failure**				
No	Reference		Reference	
Yes	0.97 (0.73–1.28)	0.804	1.08 (0.80–1.46)	0.599
**Initial therapy**				
Metformin	Reference		Reference	
DPP-4i ^e^	0.72 (0.52–0.98)	0.036	0.73 (0.53–1.00)	0.052
Repaglinide	1.29 (0.84–1.99)	0.242	1.32 (0.85–2.07)	0.216
Sulfonylureas	1.16 (0.72–1.88)	0.539	1.10 (0.68–1.80)	0.698

^a^ Crude hazard ratios (HRs) calculated using Cox regression analysis; ^b^ hazard ratios adjusted by the rest of predictors; ^c^ *p*-values less than 0.05 were considered statistically significant; ^d^ deprivation index of the area calculated according to 26 socio-economic indicators and categorized from less (Q1) to most (Q4) deprived; ^e^ dipeptidyl peptidase-4 inhibitors.
